# Poster Session I - A61 CLINICAL & PROCEDURAL PREDICTORS OF ERCP TECHNICAL SUCCESS & ADVERSE EVENTS IN PATIENTS AGED < 60 VS ≥ 60 YEARS: A RETROSPECTIVE COHORT STUDY

**DOI:** 10.1093/jcag/gwaf042.061

**Published:** 2026-02-13

**Authors:** V Mehra, K Khalaf, M Youssef, M A Bucheeri, H Li, G May, J Mosko, N Calo

**Affiliations:** Medicine, Div of Gastroenterology, St Michael’s Hospital, Toronto, ON, Canada; Medicine, Div of Gastroenterology, St Michael’s Hospital, Toronto, ON, Canada; Medicine, Div of Gastroenterology, St Michael’s Hospital, Toronto, ON, Canada; Medicine, Div of Gastroenterology, St Michael’s Hospital, Toronto, ON, Canada; Medicine, Div of Gastroenterology, St Michael’s Hospital, Toronto, ON, Canada; Medicine, Div of Gastroenterology, St Michael’s Hospital, Toronto, ON, Canada; Medicine, Div of Gastroenterology, St Michael’s Hospital, Toronto, ON, Canada; Medicine, Div of Gastroenterology, St Michael’s Hospital, Toronto, ON, Canada

## Abstract

**Background:**

According to the WHO, by 2050 the global population aged 60 years & older is projected to double, reaching ∼2.1 billion people. This demographic shift is expected to increase the demand for invasive medical procedures among older adults. Endoscopic retrograde cholangiopancreatography (ERCP) remains a cornerstone intervention in the management of pancreaticobiliary disorders & age-associated outcome data is scarce

**Aims:**

We aimed to identify determinants of ERCP technical success & adverse events among patients <60 & ≥60 years, as per WHO definition of older adults.

**Methods:**

Data from consecutive patients’ (>18) index ERCP at a large tertiary centre in Toronto, Canada from 2010-2020 were retrospectively analysed. The primary outcome was ERCP’s technical success & the secondary outcome was the occurrence of adverse events (post-ERCP pancreatitis, bleeding, perforation, or abdominal pain). Patients were stratified by age into 2 subgroups: <60 years & ≥60 years. To facilitate interpretation, a continuous age term scaled per 10-year increase was created.To identify independent predictors, univariable and multivariable logistic regression analyses were performed for the primary outcome. Collinearity was checked prior to model inclusion. Firth penalized logistic regression was applied for adverse events due to their rarity. Odds ratios (ORs) and 95% confidence intervals (CIs) were reported.

**Results:**

Among patients aged ≥60 years, altered anatomy (OR 0.17, 95% CI 0.11-0.25), use of non-duodenoscope instruments (OR 0.42, 95% CI 0.22-0.83), history of cancer (OR 0.69, 95% CI 0.54-0.90, p = 0.0048), & liver-related conditions (OR 0.60, 95% CI 0.38-0.99) were independently associated with reduced odds of technical success. Among patients aged <60 years, altered anatomy (OR 0.16, 95% CI 0.07-0.36), use of non-duodenoscope instruments (OR 0.35, 95% CI 0.14-0.90), but also presence of pancreatic disease (OR 0.56, 95% CI 0.34-0.95) was associated with lower odds of success and female sex was associated with higher odds of success as compared to males (OR 1.52, 95% CI 1.10-2.09). Among patients aged <60 years, 5.8% experienced adverse events as compared to 6.1% among those aged ≥60 years. Increased age (OR 1.26, 95% CI 1.04–1.54) & pancreatic ductal indication (OR 2.54, 95% CI 1.33–4.60) were associated with increased odds of adverse events in patients aged <60 years. Among those aged ≥60 years, general anaesthesia (OR 4.93, 95% CI 1.20–16.23) predicted higher adverse events.

**Conclusions:**

These findings highlight that both patient-related comorbidities and procedure-related factors influence ERCP success and safety differently across age strata. Recognition of these may help guide procedural planning, patient counselling and operator allocation.

**Funding Agencies:**

None

